# Supplementary material for: Research on enterprise network public opinion guiding decision-making considering crisis differentiation
Source: PLoS One. 2025 Dec 1;20(12):e0336465. doi: 10.1371/journal.pone.0336465 (PMC12668557; doi:10.1371/journal.pone.0336465)
Supplement: Attachment — Submitted filename: Response to Editors & Reviewers.doc [file pone.0336465.s002.doc]

**Original Manuscript ID:** PONE-D-25-19210

**Original Article Title: “**Research on enterprise network public opinion guiding decision-making considering crisis differentiation”

**To:** PLOS ONE Editor

Dear Editor,

Thank you for allowing a resubmission of our manuscript, with an opportunity to address the reviewers’ comments.

We are uploading (a) our point-by-point response to the comments (below) (Response to Editors and Reviewers, .doc), (b) an updated manuscript with red highlighting indicating changes (.doc) and (c) a clean updated manuscript without highlights (PDF main document).

**Re:** Response to reviewers

Dear Reviewers,

Thank you for your constructive comments concerning our manuscript entitled Research on enterprise network public opinion guiding decision-making considering crisis differentiation (ID: PONE-D-25-19210) in PLOS ONE. All those comments are very valuable and helpful for revising and improving our paper, as well as the important guiding significance to our research in future. Regardless of the results of this manuscript, we would like to express our heartfelt thanks to the Editor and all Reviewers.

After studying all the comments carefully, we have made many corrections, which we hope meet with approval. The main corrections in manuscript and the responds to the Editor’s and Reviewers' comments are as follows:

Best regards,

Jiakun Wang, Yawei Li, Xiaotong Guo, Huiying Chen

Corresponding author: Jiakun Wang

E-mail: shandongwjk@163.com

**Editor, Comments # 1:** Please ensure that your manuscript meets PLOS ONE's style requirements, including those for file naming.

**Author response:** Dear Editors, thanks for your suggestions. In the revised manuscript, we have made significant adjustments in accordance with your journal’s requirement, including abstract, authors, format, references and other contents.

**Editor, Comments # 2:** You indicated that ethical approval was not necessary for your study. We understand that the framework for ethical oversight requirements for studies of this type may differ depending on the setting and we would appreciate some further clarification regarding your research. Could you please provide further details on why your study is exempt from the need for approval and confirmation from your institutional review board or research ethics committee (e.g., in the form of a letter or email correspondence) that ethics review was not necessary for this study? Please include a copy of the correspondence as an ""Other"" file.

**Author response:** Dear editor, thank you for your concern about the ethical compliance of our research. We will provide additional explanations regarding the issue of “no ethical approval required” you raised as follows to demonstrate the ethical compliance of this study.

This paper takes practical issues in enterprises crisis management as the research object, and seeks response strategies for differentiated crisis-derived public opinion through processes such as model construction, theoretical solution, and numerical simulation. During the parameter assignment before numerical simulation, basic perspectives of participants were obtained through interviews and surveys. Throughout the survey process, we explicitly informed participants of the research objectives, data usage, and confidentiality measures, while providing an option to decline participation. The collected data were used solely for statistical analysis and were not linked to personal identity information.

**Editor, Comments # 3:** Please provide additional details regarding participant consent. In the ethics statement in the Methods and online submission information, please ensure that you have specified (1) whether consent was informed and (2) what type you obtained (for instance, written or verbal, and if verbal, how it was documented and witnessed). If your study included minors, state whether you obtained consent from parents or guardians. If the need for consent was waived by the ethics committee, please include this information. If you are reporting a retrospective study of medical records or archived samples, please ensure that you have discussed whether all data were fully anonymized before you accessed them and/or whether the IRB or ethics committee waived the requirement for informed consent. If patients provided informed written consent to have data from their medical records used in research, please include this information.

**Author response:** Dear editor, in the previously submitted manuscripts, this paper involved three authors, namely, Wang Jiakun (me), Guo Xiaotong and Chen Huiying. Guo Xiaotong is my Postgraduate student, mainly responsible for paper writing and numerical simulation.

While when we received the notice of paper revision on 9^th^ July, the second author (Guo Xiaotong) has already graduated with a master’s degree and was undergoing closed work training. Therefore, during the revision process of this manuscript, apart me and the third author (Chen Huiying), another Postgraduate student of mine (namely Li Yawei) also actively participated, ensuring the quality and progress of this revision.

After soliciting the opinions of all authors of the original manuscript (the three authors’ signature is shown in the following figure), we hope to add author (Li Yawei) and adjust the order of authorship to:

Authors Contribution:

Methodology: Jiakun Wang; Xiaotong Guo

Data curation: Jiakun Wang, Huiying Chen

Visualization & Validation: Jiakun Wang, Yawei Li, Xiaotong Guo

Writing original draft: Jiakun Wang, Xiaotong Guo

Writing review & editing: Jiakun Wang, Yawei Li

**If the above adjustments do not meet your journal’s requirement, please inform us promptly without hesitation.**

**Editor, Comments # 4:** Please note that funding information should not appear in any section or other areas of your manuscript. We will only publish funding information present in the Funding Statement section of the online submission form. Please remove any funding-related text from the manuscript.

**Author response:** Dear editor, thanks for your suggestion. According to your suggestion, we have deleted all the funding information in the revised manuscript.

**Editor, Comments # 5:** Thank you for stating the following financial disclosure: “Natural Science Foundation of Shandong Province (ZR2024MG049; ZR2021QG035) and Shandong Social Science Planning and Research Project (22DGLJ27)” Please state what role the funders took in the study. If the funders had no role, please state: "The funders had no role in study design, data collection and analysis, decision to publish, or preparation of the manuscript."

**Author response:** Dear editor, thanks for your comments. Regarding the funding support information listed in the original paper, the following explanations are provided:

Natural Science Foundation of Shandong Province (ZR2024MG049), Research on modeling and early warning of information propagation on difference-related topics in cyberspace empowered by digital intelligence technology; Recipient of funding: Wang Jiakun.

Natural Science Foundation of Shandong Province (ZR2021QG035), Research on the co-evolution of public opinion propagation and online social network structure driven by big data; Recipient of funding: Wang Jiakun.

Shandong Social Science Planning and Research Project (22DGLJ27), Research on public opinion analysis governance systems under all media era; Recipient of funding: Wang Jiakun. This project has been completed, so we have removed it from this manuscript.

Shandong Province Youth Entrepreneurship and Technology Support Program for Higher Education Institutions (2024KJH066). Wang Jiakun is a major participant, so we hope to add this project to this manuscript.

**Editor, Comments # 6:** We note that your Data Availability Statement is currently as follows: All relevant data are within the manuscript and in Supporting Information files.

**Author response:** Dear editor, thanks for your comments. The dataset used in the paper is mainly reflected in the parameter assignment part before numerical simulation. The parameter assignment process has been described in detail in the revised draft and specific parameter assignments have been provided. Therefore, we did not submit it in your journal's system.

**Reviewer #1, Comments # 1:** The first suggestion that I'd like to make to the authors would be to discuss in more depth the main choice of parameters in section 5 given by the mentioned 17 experts, for overall clarity and interpretability of the current results. Moreover, it would be interesting to see a discussion focused on the changes to the dynamics of each scenario with different values set to these parameters.

**Author response:** Dear reviewer, thank you for your professional suggestions. Indeed, as you mentioned, in the parameter definition section of the original manuscript, our explanation process was incomplete. Therefore, we are deeply sorry for the inconvenience caused to you. In the revised manuscript, we have provided a detailed description of the parameter definition stage, as follows.

To ensure the accuracy of the experimental results, we introduced the following three steps for setting the parameters. Firstly, based on research results [26-27] and actual data, the parameters were assigned as reference for subsequent steps. Then, a total of 17 experts, including professors, associate professors, and doctors in the field of public opinion and emergence management, were selected to investigate the parameters of the initial step. These experts can choose to agree with the assignment or provide their own opinions and update the results. Finally, a symposium involving 9 experts was held to fully discuss the rationality of the updated parameters until more than half of the experts approved, and the final assignment was determined. The 17experts all expressed their agreement to make the data public and signed to confirm. The main dimensionless parameters were set as follows:

$\eta_{M}=\text{15}$,$\eta_{N}=\text{10}$,$p=0\text{.}9$,$\mu=0\text{.}9$,$\varepsilon_{M}=2$,$\varepsilon_{N}=1\text{.}5$

$\lambda_{M}=0\text{.}9$,$\lambda_{N}=0\text{.}7$,$R_{M0}=0\text{.}5$,$R_{N0}=0\text{.}3$,$\omega=1\text{.}9$,$\theta=3$

The above modifications are marked red in Section 5 in the revised manuscript, as shown in the following screenshot.

**Reviewer #1, Comments # 2:** Also, even having well drawn conclusions from numeric simulations, and the parameters used having a been chosen by field experts, the manuscript extend those results directly to recommendations to real enterprises, when no real data was used to support these claims. I believe that it would suffice to reformulate the text to take that into consideration, and leave only suggestions that such conclusions can be applied to real world scenarios.

**Author response:** Dear reviewer, thank you very much for your professional comments. As you mentioned, this paper simulated real-life scenarios and theoretically discussed the strategies and approaches that enterprises should adopt in dealing with different types of crises by introducing differential game models. As for the feasibility and interpretability of the experimental results, we did not compare them with actual data.

On the one hand, for theoretical modeling and analysis papers, it is rather difficult to find real data for comparison of experimental results. And you have also pointed this opinion in your comments. On the other hand, to ensure the interpretability of the theoretical model construction and simulation analysis, we have tired to associate the theoretical model with management practice as much as possible from the following aspects.

Firstly, regarding the explanation of the topic selection, in the Introduction Section, we have provided an explanation based on the actual problems faced by enterprises to introduce the feasibility and necessity of the research on this topic.

Secondly, for the classification of crisis types, we also classify them based on actual cased to ensure the accuracy of this classification and added the introduction of four types of enterprises crises.

| Crisis type | Characteristics | Representative events | Frequencies* | Causes |
| --- | --- | --- | --- | --- |
| Product-type | Directly related to consumer rights, a “disaster area” in crisis | Samsung Note7 explosion (2016); The BMW MINI Ice Cream Incident (2023) | 35%-40% | Direct contact with consumers makes quality issues or service loopholes more likely to be exposed by the media |
| Values-type | Society’s expectations for enterprises’ responsibility have risen | The Starbucks Racial Discrimination Incident (2018); The D&G Insulting China Incident (2018) | 15%-20% | The popularization of ESG concepts has drawn much attention to controversial events |
| Internal management-type | As enterprises expand in scale and supervision becomes stricter, the frequency of outbreaks increases | Luckin Coffee's financial fraud (2020)’ Internet Layoff Wave (2023 | 20%-25% | The expansion of an enterprise's scale does not match its management capabilities |
| Marketing-type | from an occasional occurrence to the norm. | Erke (2021); Durex's marketing failure (2018) | 25%-30% | Social media amplifies the spreading effect |

Notes: The data on the frequency of crisis occurrence is derived from statistical cases of enterprises crises in the past 10 years.

Thirdly, in the Numerical Simulation Section, for the definition of experimental parameters, we also tried to determine them based on real data and expert arguments as much as possible to ensure the accuracy the parameter assignment and the accuracy of experimental results.

Lastly, for the interpretation of experimental results, we conducted a descriptive statistical analysis of experiment results to tell authors what the results are? Then, we explained and clarified the results in combination with management practices, telling authors what the reasons behind the results are? Finally, based on experimental results and interpretations, we provided feasible countermeasures and suggestion for management practices.

The above modifications are marked red in Introduction, Crisis classification, Numerical analysis. Some screenshots are shown as follows.

**Reviewer #1, Comments # 3:** Lastly, I think figures could benefit from larger font sizes, to improve readability. Figure 4 specifically should also set the z axis in the same scale for all panels, that way allowing for a better comparison between variables.

**Author response:** Dear reviewer, thank you very much for your professional comments. Indeed, as you mentioned, the presentation of picture in the original manuscript was not very clear. Therefore, we are deeply sorry for the inconvenience caused to you. In response to this comment, we have modified all pictures in the revised manuscript to ensure their clear presentation. In addition, we have also provided detailed definitions and explanations for the meanings represented by the coordinate axes of each picture. Partial figures are shown as follows.

**Reviewer #1, Comments # 4:** Overall, future research with this methodology could attempt to study recurring crisis of confidence to a given enterprise, and how public opinion would be affected by these repeating events, which in turn would help understand the behavior of decade-spawning institutions and also allow for a study of stability of those systems.

**Author response:** Dear reviewer, thank you very much for your affirmation of the topic selection and research paradigm of this manuscript. As you mentioned, the distribution of benefits among key stakeholders, crisis prediction and the recurrence of crisis leading to diminishing marginal utility will significantly impact the guiding effect of public opinion derived from crisis. These areas require further in-depth research in the future. The above modifications are described in Conclusion section and shown as following screenshot.

**Reviewer #2, Comments # 1:** A thorough revision of the language is necessary. Some sentences are unclear, and there are many typos. For example in the Literature Review: ‘the mechanism of public opinion affect mechanism, […]’ , or at the end of Sec. 3: ‘Enterprises provide relevant guidance and subsidy measures to media to encourage them to spread truthful and favourable information to enterprises to clarify the truth.’ Or, again, in Sec. 4.1: ‘The Hamilton-Jacobi-Bellman (HJB) equation is used to solve the model, drawing on the approach outlined in.’(?).

**Author response:** Dear review, thank you very much for your professional and detailed comments. Indeed, as you mentioned, it is highly necessary to make a thorough language revision to the original manuscript. And we are deeply sorry for the inconvenience caused to you. To facilitate reading of this paper, we have carefully examined each word, each sentence and each punctuation mark, and modified them to the correct expression in revised manuscript. Considering that the above content has been modified too much, it is not marked red in the revised manuscript.

**Reviewer #2, Comments # 2:** Some acronyms are never defined throughout the paper, for example, OSN, SNP, WP and many others.

**Author response:** Dear review, thank you very much for your comments. Indeed, due to our carelessness, there are many abbreviations in the original manuscript that do not provide their full names, which is completely contrary to academic norms. We are also deeply sorry for this. In the revised manuscript, we have carefully examined all the abbreviations in this paper to ensure that they presented their full names when they first appeared. The screenshots of some content modifications are shown as follows.

**Reviewer #2, Comments # 3:** The crisis classification is clear and well-described. However, the model section requires a complete revision. In particular, the "Basic Assumption" section is merely a list of definitions of the quantities involved in the differential game. It should be rewritten as a section that properly illustrates the basic assumptions, not as a mere list pf quantities. generally, indexes on quantities are generally so small that they are difficult to read. Some parameters are also never defined, such as the eta_M and eta_N.

**Author response:** Dear review, thank you very much for your affirmation of our research content and your professional comments. As you mentioned, in the Basic Assumption section of the original manuscript, we wrote it too simply and mainly focused on the introduction and definition of variables, with insufficient description of the connection between the introduction of variables and the problem. Based on your comments, we have rewritten the 3.3 Basic Assumptions section in the revised manuscript, focusing on introducing the purpose of the variable introduction and its connection with the research questions of this paper. In particular, we have explained and clarified the meanings of all variables appearing in the assumptions to facilitate readers' better understanding of this paper. The above modifications are revised and marked red in Section 3.3 Basic Assumptions. The screenshots of some content modifications are shown as follows.

**Reviewer #2, Comments # 4:** I understand that C_Mi(t), that is the cost of reporting by the media at the time of the type i crisis, is a quadratic function in the corresponding effort degree. This is a common assumption indeed. So I can understand what the cost for media is for producing and spread information but I don’t understand what kind of cost is involved for netizens, who mainly are users of social networks, I suppose. Could the author please better clarify what kind of cost they are referring to?

**Author response:** Dear review, thank you very much for your professional comments. In the original manuscript, although we have defined and functionally represented media and netizens costs involved in the spreading process of public opinion, the underlying logic is indeed not clearly explained. We are deeply sorry for this. Based on your feedback, we have provided a detailed description of their costs for in the revised manuscript, as shown follows.

For media, what they need to decide is the degree of their efforts to report and spread positive public opinion on social platforms after the occurrence of crisis *i,* denoted by $E_{\text{Mi}}\left( t \right)$. During the reporting process of media, their costs are mainly reflected in aspects such as tracking and reporting on events and uncovering the truth, which is a convex function of their efforts, represented by $C_{\text{Mi}}\left( t \right)=\frac{\eta_{M}}{2}(1-U_{i1})E_{\text{Mi}}^{2}\left( t \right)$. Among them, $\eta_{M}$ is the coefficient between the degree of media’s effort and their cost. When enterprises didn’t provide subsidy to media, their cost function becomes $C_{\text{Mi}}\left( t \right)=\frac{\eta_{M}}{2}E_{\text{Mi}}^{2}\left( t \right)$.

For netizens, they need to decide the degree of their efforts to spreading positive public opinion in social platforms after crisis $i$, represented by $E_{\text{Ni}}\left( t \right)$. And during the spreading process of public opinion, the costs for netizens mainly relies in the time and energy they spend paying attention to, collecting, organizing and spreading, which is also a convex function of their efforts, denoted by $C_{\text{Ni}}\left( t \right)=\frac{\eta_{N}}{2}(1-U_{i2})E_{\text{Ni}}^{2}\left( t \right)$. Among them, $\eta_{N}$ is the coefficient between the degree of netizens’ effort and their cost. When enterprises didn’t provide subsidy to the,, their cost function becomes $C_{\text{Ni}}\left( t \right)=\frac{\eta_{N}}{2}E_{\text{Ni}}^{2}\left( t \right)$.

**Reviewer #2, Comments # 5:** The authors propose a differential equation (1) for the credibility of the media. Is this equation found in the literature, and why was it chosen? Is it universal? I would also like to emphasize the role and meaning of the omega coefficient, which represents implicit guidance, as well as the difference between unguided and explicitly guided types on the left side of Eq. (1).

**Author response:** Dear review, thank you very much for your professional comments. In the original manuscript, we introduced Eq. (1-2) to describe the credibility of media and netizens, and gave the differential equation for the change in their credibility. Indeed, as you mentioned, our explanations of the Eq. (1-2) are insufficient and we are sorry for the inconvenience caused to you. In response to your comments, we have provided a detailed introduction and clarification of Eq. (1-2) in the revised manuscript.

Firstly, we introduced the credibility of media and netizens to represent their influence on social platforms, and then discuss the relationship between it and the spreading scope of public opinion. Obviously, when focusing on the public opinion derived from a certain type of enterprises crisis, the credibility of media and netizens on social platforms will increase with the improvement of their efforts in spreading or reporting this event, and will decrease over time, which can be regarded as a natural attenuation phenomenon. Based on the current relatively mature research paradigm [23-25], we introduce a credibility differential equation to represent its variation over time, that is,

$\dot{R}_{M}\left( t \right)=\sum_{i=1}^{n} \varepsilon_{M}E_{\text{Mi}}-{\mu R}_{M}\left( t \right)$，$\dot{R}_{M1}\left( t \right)=\sum_{i=1}^{n} \left( \varepsilon_{M}+\omega\right)E_{\text{Mi}}-{\mu R}_{M}\left( t \right)$	（1）

Secondly, after introducing the credibility of media and netizens, we hope to quantitatively represent them. In addition to their efforts and the natural attenuation effect, their credibility may also be affected by the promotion effect of enterprises. For example, enterprises can rapidly expand the spreading scope of public opinion by leveraging technologies such as algorithm recommendation and information push. Under the support of the implicit guidance of enterprises, the credibility of media and netizens will increase significantly than before. In this manuscript, $\omega$ is introduced to represent the impact of the implicit incentives of enterprises on their credibility which in turns affects the spreading scope of public opinion.

The above modifications are marked red in Section 3.3 Basic assumptions and shown as following screenshots.

**Reviewer #2, Comments # 6:** The p discount rate is defined at the end of Sec. 3 but it appears only below in the HJB Eqs (5) first.

**Author response:** Dear reviewer, regarding the discount rate (*p*), we would like to offer the following explanations.

Firstly, in differential games, the discount rate (*p*) is a key parameter used to describe the "time value" - that is, the degree to which future gains or costs depreciate relative to the current value. Its core function is to convert future benefits/costs over continuous time into current values, thereby influencing the intertemporal decision-making logic of participants.

Secondly, differential game studies the strategic interactions among multiple participants (players) over continuous time. Its core lies in describing the dynamic changes of state variables through differential equations and solving the optimal strategies of the participants through optimal control theory. During this process, "time" is a continuous variable. Due to the decision-maker's time preference (valuing immediate gains over future gains) or opportunity cost (one unit of future gains requires sacrificing current investment or consumption), future gains need to be "discounted" to their current value at a certain proportion. This is the main reason why the discount rate is introduced.

Thirdly, in this manuscript, we defined the discount rate (*p*) in Section 3.3, and it also appeared many times in the subsequent model solving process. Such as the optimal value functions for the revenue of enterprises, media and netizens, as shown in Eq. (7, 10, 13).

**Reviewer #2, Comments # 7:** Before Section 4.1, I suggest clarifying the classification of crises and strategies because there are four types of crises and four types of guidance strategies. A table or explanatory scheme would be useful to avoid any confusion.7.

**Author response:** Dear review, thank you very much for your professional comments. In the original manuscript, we introduced four types of crises and four decision-making scenarios before 4.1, but the reasons and justifies for their introduction have not been clearly explained, which may cause confusion among readers. In response to your comments, we have made key modifications in the revised manuscript from the following two aspects.

Firstly, for the classification of enterprises crises. Combining the actual cases of enterprises crises outbreaks in recent years with the existing literature [22], this paper examines the following four common types of crises. And we also provided a detailed introduction to the four types of crises and summarized their characteristics, causes, representative events and occurrence frequencies.

Secondly, for the definition of decision-making scenarios. After the occurrence of crisis, there are many strategies available for enterprises to choose. Based on the possible behaviors of enterprises in practical management, we introduced four decision-making scenarios.

- Decentralized decision-making without guidance strategy (***f***): After the occurrence of crisis, enterprises took no action, and both the media and netizens made decisions with the goal of maximizing their own interests.
- Decision-making scenarios with explicit guidance: To effectively guide public opinion after crisis, enterprises often employ strategies to encourage media and netizens to create and spread public opinion. Enterprises can provide detailed information to help media and netizens overcome challenges in verifying facts or offer subsidies to reduce their financial burden. This direct intervention and facilitation of public opinion propagation is termed the explicit guidance strategy. Under this scenario, based on whether the media and netizens can reach cooperation, this scenario can be further refined into decentralized decision-making with explicit guidance strategy (***xf***), centralized decision-making with explicit guidance strategy (***xj***).
- Decentralized decision-making with dual guidance strategy (***df***). In addition to explicit guidance, enterprises can enhance the visibility of media reports and public opinion released by netizens with the help of personalized algorithms. While obtaining traffic revenue, media and netizens can also strengthen their social recognition, that is, increasing the influence of the degree of their effort in reporting and spreading on its credibility, thus forming dual guidance scenario. Besides, according to the general principle of differential games [23], centralized decision-making model in an unconstrained environment typically yields higher system benefits than decentralized decision-making. However, considering the complex factors such as information asymmetry in real world, decentralized decision-making scenarios are more widely applied compared to the centralized decision-making scenarios in ideal states.

The above modifications are marked red in Section 3.2 Crisis classification and Section 4, shown as follows.

**Reviewer #2, Comments # 8:** Fix the name of the author Jorgensena in the text and bibliography.

**Author response:** Dear reviewer, thank you for pointing out the mistake in the citation of this paper, and we are very sorry. In the revised manuscript, we have carefully checked all the citations and references to ensure that there are no more basic mistakes such as spelling of names and titles.

**Reviewer #2, Comments # 9:** In Eq. (5) and the similar equations that follow, I would explicitly state the variables. What variables does the maximum apply to?

**Author response:** Dear reviewer, for Eq. (5) and the following equations, our detailed explanations are as follows.

Taking Eq. (5) as an example, it presents the media’s revenue function in decentralized decision-making scenario throughout the entire decision-making cycle, and discharged the total revenue in the future time to the current moment with the help of the discount rate. Based on this revenue function, media will make decisions to maximize their revenues, and their decision variable is the degree of effort they make in reporting public opinion. And the solution is shown in Eq. (6). This method is applicable to the determination of all stakeholders’ strategies. After solving all stakeholders’ strategies (such as the degree of effort of media and netizens, the subsidy coefficient of enterprises), based on the basic assumptions of Section 3.3, we can provide the analytical expressions of all variables.

**Reviewer #2, Comments # 10:** In Eq. (7) I don’t understand the dependence on time t of the right hand side. Is there no integral over time?

**Author response:** Dear reviewer, thank you for pointing out the mistakes in the Eq. (7) and the following similar equations. Indeed, as you mentioned, Eq. (7) shows the optimal value functions for the revenue of media and netizens, aiming to describe the “optimal expected revenue” of all stakeholders in a continuous-time dynamic game, which is the maximum discounted total revenue that one stakeholder can obtain. Essentially, it is a quantitative manifestation of the equilibrium state of the game, which is the sum of the revenue obtained by the stakeholders throughout the entire game cycle. We have made corrections in the revised manuscript.

**Reviewer #2, Comments # 11:** The comments for each type of strategy in Sec 4.1, and followings, limit to the positive or negative correlations between the optimal decision variables and other parameters. I would expect some more interpretation of the optimal values.

**Author response:** Dear review, thank you very much for your professional comments. Indeed, as you mentioned, in the original manuscript where we interpret the theoretical analysis results, we only remain at the surface level of description and analysis, lacking in-depth interpretation and discussion. In response to your comments, we have revised the interpretation section of the theoretical results.

Firstly, for the theoretical solution results in the four scenarios, we refined the description and analysis of the theoretical results based on the original manuscript. At the same time, some identical content was deleted to avoid making the content overly lengthy.

Secondly, we rewrote Section 4.5: Comparison and Analysis. On the one hand, we compared the theoretical results of the above four scenarios and summarized the results of the theoretical analysis. Such as, (1) In the scenario without guidance, media and netizens have the lowest degree of effort, and with the intervention of enterprises guidance strategies, their efforts have improved. (2) Media and netizens have higher degree of effort in centralized decision-making scenario, while enterprises need to pay higher subsidy coefficient in decentralized decision-making scenario. (3) In centralized decision-making scenario, the credibility of media (netizens), the attention and spreading range of public opinion are all higher than those in decentralized decision-making scenario. (4) The implicit guidance coefficient plays a key role when comparing the strategic choices of media and netizens in ***xj*** and ***df*** scenarios.

On the other hand, for the differences in theoretical results under the four scenarios, we have provided detailed explanations and clarifications in combination with our understanding of this issue. Such as, for the inference (2), in decentralized decision-making scenario, media and netizens “fight on their own”, pursuing the best benefits for themselves, and fail to collaborate. This leads to both stakeholders paying a relatively high cos for the propagation of public opinion, which in turn results in a lower degree of effort and a poor effect in guiding public opinion. In centralized decision-making scenario, for the sub-game system formed by media and netizens, in addition to enjoying subsidies provided by enterprises, their cooperation further reduces the cost of public opinion propagation. While expanding the spreading scope of public opinion, the improvement of their credibility also prompts them to actively participate in the cooperation process and have more effort. Besides, the subsidy for media and netizens provided by enterprises is relatively low, i.e., enterprises can obtain better public opinion guidance effects with a lower cost. This is also since the cooperation of the media and netizens reduces the cost of public opinion reporting (spreading), further stimulating the enthusiasm of both stakeholders.

The above modifications are marked red in Section 4.

**Reviewer #2, Comments # 12:** In Corollary 2 they appear for the first time these terms for omega: stealthy bootstrapping coefficient and the invisible bootstrap coefficient

**Author response:** Dear reviewer, we are deeply sorry for the inaccurate description of the professional terms. In the revised manuscript, we have unified the frequently used professional terms and provided detailed introduction to them.

**Reviewer #2, Comments # 13:** In Sec. 5, the authors justify the selection of parameter values based on the opinions of several experts. I personally have no reason not to trust their expertise, but it would be nice to find some real motivation for the values of at least some of the involved parameters.

**Author response:** Dear reviewer, thank you for your professional suggestions. Indeed, as you mentioned, in the parameter definition section of the original draft, our explanation process was incomplete. Therefore, we are deeply sorry for the inconvenience caused to you. In the revised draft, we have provided a detailed description of the parameter definition stage, as follows.

To ensure the accuracy of the experimental results, we introduced the following three steps for setting the parameters. Firstly, based on research results [26-27] and actual data, the parameters were assigned as reference for subsequent steps. Then, a total of 17 experts, including professors, associate professors, and doctors in the field of public opinion and emergence management, were selected to investigate the parameters of the initial step. These experts can choose to agree with the assignment or provide their own opinions and update the results. Finally, a symposium involving 9 experts was held to fully discuss the rationality of the updated parameters until more than half of the experts approved, and the final assignment was determined. All experts expressed their agreement to make the data public and signed to confirm. The main dimensionless parameters were set as follows:

$\eta_{M}=\text{15}$,$\eta_{N}=\text{10}$,$p=0\text{.}9$,$\mu=0\text{.}9$,$\varepsilon_{M}=2$,$\varepsilon_{N}=1\text{.}5$

$\lambda_{M}=0\text{.}9$,$\lambda_{N}=0\text{.}7$,$R_{M0}=0\text{.}5$,$R_{N0}=0\text{.}3$,$\omega=1\text{.}9$,$\theta=3$

The above modifications are marked red in Section 5 in the revised manuscript, shown as in the following screenshot.

**Reviewer #2, Comments # 14:** The captions of the figures must be improved. For example, the meaning of the four curves in Fig. 2 should be explained. Moreover, the readability of the legends in the figures should be increased.

**Author response:** Dear reviewer, thank you very much for your professional comments. Indeed, as you mentioned, the presentation of picture in the original manuscript was not very clear. Therefore, we are deeply sorry for the inconvenience caused to you. In response to this comment, we have modified all pictures in the revised manuscript to ensure their clear presentation. In addition, we have also provided detailed definitions and explanations for the meanings represented by the coordinate axes of each picture.

**Reviewer #2, Comments # 15:** In Sec. 5.3, please clarify why the authors only determine g₂ and then assign the other gi values according to their priority. What does this mean? Below, I see three figures for three of them. How are these figures obtained?

**Author response:** Dear reviewer, thanks for your professional comments. Indeed, as you mentioned, in Section 5.3, The proportion of resources allocation for guiding public opinion derived from four types of crises under the limited resources was not introduced very clearly in the original manuscript, which led to your misunderstanding. For this point, we would like to explain and clarify from the following aspects.

Firstly, as can be seen from the conclusion in Section 5.2, in ***df*** decision-making scenario, the evolution trends of different types of crises follow similar pattern. However, different types of crises bring differentiated benefits to enterprises during the responding process. Such as shown in Figure 2(c), in the responding process to the four types of crises, the order of the benefits brought by each type of crisis to enterprises from largest to smallest is: the second type (Values-type) crises, the fourth type (Marketing-type) crises, the first type (Product-type) crises and the third type (Internal management-type). The above conclusion provides an important reference for enterprises to deal with crises. In other words, the experimental results and analyses in Section 5.2 have helped enterprises determine the priority management order for guiding public opinion, i.e., enterprises should prioritize the values-type crises, followed by the product-type crises, marketing-type crises, and internal management-type crises.

Secondly, after determining the sequence of the enterprises’ crisis response, our approach to determining the allocation ration of various crisis response resources is as follows. If all four types of crises occur simultaneously, according to the crisis response sequence, enterprises should deal with Values-type crises first. Supposing the enterprises resources are “1”, then the response resources are allocated from 0 to 1. Taking the allocation ratio as the independent variable and the overall enterprises revenue as the observation variable, the resource allocation ratio obtained by Values-type crises can be determined when the overall enterprises revenue is the largest. The numerical results show that when the allocation ration of the Value-types crises is approximately 60%, the overall revenue of enterprises is the largest. That is, we have determined the proportion of resources to deal with the Values-type crises is 60%.

Next, we need to determine the proportion of resources allocated for the Marketing-type crises. Supposing that enterprises have allocated 60% of their governance resources to the Values-type crises, the total amount of their resources drops from the original “1” to the current “0.4”. Then, following the similar approach, taking the ratio of resources obtained by the Marketing-type crises as the independent variable (ranging from 0 to 0.4), and the overall revenue of enterprises as the dependent variable, the changes in the overall enterprise revenue under different allocation ratios were numerically simulated, as shown in Figure 3b. The experimental results show that when the proportion of governance resources obtained for the Marketing-type crises is approximately 28%, the overall benefit of enterprise is the greatest. Subsequently, it is determined that the proportion of resources for responding to the Marketing-type crises is 28%.

Then, we will continue to determine the proportion of resources allocated to the Product-type crises. At this point, the total amount of enterprises governance resources has dropped from the original “1” to the current “0.12”. Following the similar process approach, taking the proportion of resources allocated to the Product-type crises as the independent variable, the optimal resources allocation ratio can be determined through observing the changing process of the overall revenue of enterprises, as shown in Figure 3c. Then, we found the resource allocation ratio to the Product-type crises is approximately 8.4%.

Finally, enterprises have consumed 96.4% of their governance resources, and the remaining 3.6% of their resources will be allocated to the Internal Management-type crises.

Regarding the issue of determining the proportion of resource allocation for different types of crises, we have adopted an approach for allocating resources one by one to solve this problem. However, the above-mentioned handling approach assumes that the handling processes of different types of crises are relatively independent, that is, different types of crises do not interfere with or influence each other during the handling process. To break this basic assumption, in the future research, we plan to take the allocation rations to four types of crises (x, y, z, 1-x-y-z) as independent variable and the overall revenue of enterprises as the dependent variable. Then, with the help of numerical simulations and machine learning algorithms, we can also determine the optimal allocation ration combination. And the above content was also described in the manuscript’s outlook.

The above modifications are shown as following screenshots.

In addition to the above modifications, we also revised the description of the article in order to improve the quality of this paper and facilitate readers to read. And to ensure the accessibility of the references, we have updated the references in the original manuscript to guarantee their accessibility to the greatest extent. Partial references as shown follows.

*Zhao X, Huang Q, Xing B, Zhang H. The motivating effect of positive social emotions on digital transformation of manufacturing enterprises–evidence from financial news and public opinion. Technology Analysis & Strategic Management. 2024, 1-15. DOI: 10.1080/09537325.2024.2424432*

*Qiu Z, Yuan X, Yin Y. Research on social governance of network public opinion: An evolutionary game model, Discrete Dynamics in Nature and Society. 2023, 8530530. DOI: 10.1155/2023/8530530.*

*Venkateswarlu Y, Baskar K, Wongchai A, Shankar V, Carranza C. An efficient outlier detection with deep learning-based financial crisis prediction model in big data environment. Computational Intelligence and Neuroscience. 2022, 4948947. DOI: 10.1155/2022/4948947.*

*Van N, Kommers P, Van H, Joost V. Damaged corporate reputation: can celebrity tweets repair it? Computers in Human Behavior. 2014, 36(0): 308-315. DOI: 10.1016/j.chb.2014.03.056.*

*Awad T, Asmaa A, Maan T, Waleed A. Dynamic analysis of competitive marketing strategies using differential game models and Runge–Kutta solutions. Partial Differential Equations in Applied Mathematics. 2025, 14: 101156. DOI: 10.1016/j.padiff.2025.101156.*

*Momeni B, Mohebbi S. Differential Game Theoretic Models for Designing Water Conservation Incentives. Decision Analysis, 2025. DOI: 10.1287/deca.2024.0208.*

We have tried our best to improve the quality of this manuscript and made some changes in the original manuscript. These changes will not influence the content and framework of the paper. And here we do not list all changes in the original manuscript. And if you have any questions about this paper, please don't hesitate to let us know.

We appreciate for Editors/Reviewers' warm work earnestly again, and hope that the above corrections will meet with approval. And once again, thank you very much for your excellent, professional comments and suggestions.

Thank you and best regards.

Yours sincerely,

Jiakun Wang, Yawei Li, Xiaotong Guo, Huiying Chen

Corresponding author: Jiakun Wang

E-mail: shandongwjk@163.com
