# Supplementary material for: Research on enterprise network public opinion guiding decision-making considering crisis differentiation
Source: PLoS One. 2025 Dec 1;20(12):e0336465. doi: 10.1371/journal.pone.0336465 (PMC12668557; doi:10.1371/journal.pone.0336465)
Supplement: Attachment — Submitted filename: Response_to_Editors_&_Reviewers_auresp_2.doc [file pone.0336465.s003.doc]

**Original Manuscript ID:** PONE-D-25-19210R1

**Original Article Title: “**Research on enterprise network public opinion guiding decision-making considering crisis differentiation”

**To:** PLOS ONE Editor

Dear Editor,

Thank you for allowing a resubmission of our manuscript, with an opportunity to address the reviewers’ comments.

First, in accordance with your instructions, we have carefully checked all the references listed in this manuscript one by one. All the references are complete and correct, and they have not been retracted. Therefore, we have not made any changes to the references.

Then, we are uploading (a) our point-by-point response to the comments (below) (Response to Editors and Reviewers, .doc), (b) an updated manuscript with red highlighting indicating changes (Revised Manuscript with Track Changes) (.doc) and (c) a clean updated manuscript without highlights (Manuscript) (.doc).

**Re:** Response to reviewers

Dear Reviewers,

Thank you for your affirmation of our manuscript entitled Research on enterprise network public opinion guiding decision-making considering crisis differentiation (ID: PONE-D-25-19210R1) and the recognition of our revision during the previous round of revision. All those comments are very valuable and helpful for revising and improving our paper, as well as the important guiding significance to our research in future.

After studying the comments carefully, we have some corrections, which we hope meet with approval. The main corrections in manuscript and the responds to the Editor’s and Reviewers' comments are as follows:

Best regards,

Jiakun Wang, Yawei Li, Xiaotong Guo, Huiying Chen

Corresponding author: Jiakun Wang

E-mail: shandongwjk@163.com

**Reviewer #1, Comments # 1:** I just want to point out that the caption of Fig. 1. c) might be a typo. Shouldn't it be "credibility of the netizens", as it was in the original manuscript? I've personally not found other similar disparities while reading this revised version.

**Author response:** Dear reviewer, thank you for pointing out this mistake. Indeed, as you said, the Fig. 1(c) describes the evolution trend netizens’ credibility. We have made corrections in the revised draft and carefully checked the remaining figures to ensure that there no similar errors. Finally, we still apologized to you for our carelessness.

The revised Fig. 1 are shown as follows.

**Figure 1. The evolution trends of various variables under four scenarios**

We appreciate for Editors/Reviewers' warm work earnestly again, and hope that the above corrections will meet with approval. Once again, thank you very much for your excellent, professional comments and suggestions. And if you have any questions about this paper, please don't hesitate to let us know.

Thank you and best regards.

Yours sincerely,

Jiakun Wang, Yawei Li, Xiaotong Guo, Huiying Chen

Corresponding author: Jiakun Wang

E-mail: shandongwjk@163.com
